# Challenges to continuity of care in volunteer-integrated services for older adults: a mixed-methods study in urban China

**DOI:** 10.1186/s12877-026-07115-4

**Published:** 2026-02-11

**Authors:** Mingzhu Yang, Shuang Chai, Jinsong Pan, Junxin Li, Linnan Li, Janiece L. Taylor

**Affiliations:** 1 Shanghai General Hospital, Shanghai Jiao Tong University School of Medicine, Shanghai, China; 2 Johns Hopkins School of Nursing, Baltimore, MD USA

**Keywords:** Older adults, Volunteer-supported eldercare services, Continuity of care, Long-Term care insurance, Digital literacy, Mixed-methods, Community-based services, China

## Abstract

**Background:**

China’s rapidly aging urban population has intensified demand for sustainable community-based eldercare. Volunteer-supported eldercare services are increasingly promoted as complements to formal care, yet their contribution to functional, relational, informational, and managerial continuity remains insufficiently understood. This study examined systemic barriers to continuity and explored strategies for embedding volunteers within integrated eldercare systems.

**Methods:**

An explanatory sequential mixed-methods design Quantitative → Qualitative (QUAN→QUAL) was conducted in Shanghai’s most aged districts, Hongkou and Huangpu. A cross-sectional survey of 880 older adults assessed service willingness, utilization, and predictors of dissatisfaction using binary logistic regression. Subsequently, 21 in-depth interviews with older adults, volunteers, and Red Cross staff explored mechanisms underlying observed discontinuities. Quantitative and qualitative findings were integrated using the Pillar Integration Process and mapped onto Haggerty’s four-dimensional continuity framework.

**Results:**

Although 96.3% of respondents expressed willingness to use volunteer-supported eldercare services, only 46.5% had done so. Service dissatisfaction was strongly associated with frequent volunteer turnover (aOR = 2.31; 95% CI: 1.83–3.14) and digital illiteracy, particularly among adults aged ≥ 80 years (89.3%). Integrated analyses identified four interrelated barriers undermining continuity: restrictive Long-Term Care Insurance (LTCI) eligibility excluding moderately disabled older adults; unstable volunteer–client relationships; digital and informational gaps compromising coordination and safety; and fragmented governance limiting cross-sector collaboration.

**Conclusions:**

Bridging the willingness–utilization gap requires systemic reform across policy, organizational, service, and individual levels. Priorities include expanding LTCI eligibility, formalizing volunteer roles within interdisciplinary teams, establishing navigation mechanisms for unmet “grey-zone” needs, and enhancing digital literacy through hybrid information systems. By extending Haggerty’s continuity framework beyond clinical care, this study illustrates how volunteers can be positioned as integrated extensions of professional teams—supported by stable governance and interoperable information platforms—to enable person-centered, sustainable eldercare in super-aged urban settings.

**Supplementary Information:**

The online version contains supplementary material available at 10.1186/s12877-026-07115-4.

## Introduction

Global population ageing is accelerating worldwide. By 2050, adults aged 65 years and older are projected to comprise 16.0% of the global population—approximately 1.5 billion people—up from 9.3% in 2020 [1]. This demographic shift places growing pressure on health and social care systems, particularly in maintaining the continuity and quality of eldercare. In response, volunteer-supported eldercare services—unpaid, non-clinical activities such as companionship and assistance with activities of daily living (ADLs)—have increasingly been promoted as complements to formal care, with the potential to enhance system resilience. Evidence from nursing practice suggests that when role boundaries and supervision are clearly defined, volunteer involvement is perceived as a safe and valuable addition to professional services [2]. Nevertheless, the institutional integration of volunteers into structured eldercare systems remains uneven across countries.

International experience illustrates how formalized volunteer integration can strengthen continuity in community-based eldercare. In the United Kingdom, the Royal Voluntary Service’s *Good Neighbors* program enhances relational continuity through regular home visits that reduce social isolation [3]. Australia’s Community Visitors Scheme employs federally funded coordination mechanisms to promote social participation, representing a structured approach to managerial continuity [4]. Similarly, municipality-backed initiatives in Nordic countries embed volunteers within local governance arrangements, enabling them to function as stable extensions of home-based care [5]. Collectively, these models suggest that volunteer-supported services are most effective when institutionally embedded, ensuring that care is not episodic but sustained across functional and relational dimensions.

Translating such approaches to China requires careful consideration of a distinctive policy and demographic context. Population ageing is advancing rapidly, particularly in large metropolitan areas; by late 2024, older adults accounted for 37.6% of Shanghai’s population [6]. At the same time, traditional family-based caregiving has weakened as a result of urbanization and declining fertility [7–10]. Although national initiatives such as the “9073” model and the *yiyang jiehe* strategy aim to integrate health and social care [11, 12], the Long-Term Care Insurance (LTCI) program often excludes individuals with moderate disability [13]. This exclusion has created a “grey zone” of unmet needs, effectively shifting additional care responsibilities onto volunteer-supported eldercare services.

In this study, *volunteer-supported eldercare services* are defined as organized, non-clinical services delivered by semi-formal actors—such as volunteers coordinated by residents’ committees or the Red Cross—that are institutionally embedded within, and operationally coordinated with, local health and social care systems, rather than informal or episodic community participation [14]. Despite their growing prominence, the development of these services in China remains fragmented. Compared with international models, volunteer-supported eldercare services are often characterized by project-based funding and a lack of standardized operating procedures (SOPs), contributing to discontinuities in service delivery and coordination. These conditions underscore the need for systematic examination of how volunteer-supported eldercare services contribute to continuity, and where structural barriers—ranging from restrictive policy eligibility to high volunteer turnover—undermine their effective integration.

To address these issues, we adopt the four-dimensional continuity of care framework proposed by Haggerty et al. [15]. Within the context of volunteer-supported eldercare services, we conceptualize functional continuity as the alignment of services with older adults’ daily and psychosocial needs; relational continuity as the stability of trust-based relationships between volunteers and older adults; informational continuity as the effective transfer and usability of health-related information; and managerial continuity as cross-sector coordination and oversight. Although originally developed to examine clinical care pathways, this framework provides a system-level lens for analyzing how organizational arrangements, service structures, and governance mechanisms shape the integration of non-clinical, volunteer-supported eldercare services. Its multidimensional structure is particularly well suited to capturing both structural (functional and managerial) and interpersonal (relational and informational) aspects of continuity.

Existing applications of Haggerty’s framework have focused primarily on formal healthcare settings, with limited attention to volunteer-supported eldercare services. By explicitly incorporating volunteers as semi-formal actors within the continuity framework, this study extends its application beyond clinical care and addresses an important gap in continuity research. Accordingly, we employ an explanatory sequential mixed-methods design Quantitative → Qualitative (QUAN→QUAL) [16] to: (1) quantitatively identify barriers to the utilization and continuity of volunteer-supported eldercare services; (2) qualitatively explore the mechanisms underlying observed discontinuities; and (3) integrate findings to inform strategies for sustainable volunteer engagement.

Empirically, the study focuses on two central districts in Shanghai—Hongkou and Huangpu—which are among the most aged urban districts in the city, each with an older population proportion exceeding 44% [6]. Their high population density and well-established community governance structures make them particularly suitable settings for examining continuity challenges and the institutional integration of volunteer-supported eldercare services in urban China.

## Method

### Study design and Mixed-Methods integration

This study adopted an explanatory sequential mixed-methods design (QUAN→QUAL) [16]. The design was selected to first quantify the prevalence and distribution of barriers to continuity of care—defined here as the coordinated integration of volunteer-supported eldercare services delivered by semi-formal actors and aligned with formal health and social care systems—and subsequently to explore the mechanisms underlying these barriers through qualitative inquiry.

The quantitative phase comprised a cross-sectional survey of 880 community-dwelling older adults, followed by a qualitative phase involving 21 in-depth interviews. Integration of findings was guided by the Pillar Integration Process (PIP) framework [16], which involved: (1) aligning results across datasets; (2) identifying convergence, divergence, and complementarity; (3) generating meta-inferences; and (4) mapping integrated findings onto Haggerty et al.’s four dimensions of continuity—functional, relational, informational, and managerial. Joint displays were used as analytic tools to enhance transparency and support mechanism-oriented synthesis [17]. Reporting adhered to the Standards for Reporting Qualitative Research (SRQR) [18] and the Good Reporting of a Mixed Methods Study (GRAMMS) guidelines [19].

### Study setting

Shanghai was selected as the study setting due to its advanced population ageing and early adoption of community-based eldercare reforms. Two central districts—Hongkou and Huangpu—were purposively selected, both reporting ageing rates exceeding 44% [6]. While sharing high population density and relatively mature community governance structures, the districts differ in organizational delivery models: Huangpu has more integrated medical–nursing service platforms, whereas Hongkou places greater emphasis on neighborhood-based Red Cross volunteer networks [6]. Inclusion of both districts enabled examination of how local organizational arrangements shape continuity of care within volunteer-supported eldercare service systems.

### Quantitative phase

#### Participants and sampling

Between November 2023 and January 2024, a cross-sectional survey was conducted in Hongkou and Huangpu districts. Eligible participants were community-dwelling adults aged ≥ 60 years who had resided in the district for at least 12 months and were able to provide informed consent. Stratified quota sampling was applied to reflect local age distributions [6], rather than to achieve population representativeness, comprising 49.3% aged 60–69 years, 39.0% aged 70–79 years, and 11.7% aged ≥ 80 years. Based on a priori power calculations assuming 23 predictors and allowing for a 20% non-response rate, the minimum required sample size was estimated at 288–575 [20]. A total of 947 individuals were approached, yielding 880 valid responses (response rate: 92.9%).

#### Measures and operationalization

The structured questionnaire was adapted from Zhao’s validated urban eldercare needs scale [21]. Psychometric testing demonstrated satisfactory reliability and validity (content validity index = 0.95; Cronbach’s α = 0.85; split-half reliability = 0.84; test–retest reliability = 0.92; pilot *n* = 20). To ensure theory-driven operationalization, core variables were explicitly linked to Haggerty et al.’s four continuity dimensions.

Functional continuity (functional status) was assessed using the Katz Index of Activities of Daily Living (ADL) [22], categorized as fully independent (score = 6), partially dependent (scores 3–5), or fully dependent (scores ≤ 2), reflecting the alignment between service provision and older adults’ functional needs.

Relational continuity (volunteer turnover) was defined as three or more changes in assigned volunteers within a six-month period, based on prior evidence suggesting that relational continuity deteriorates beyond two replacements [23–25]. This indicator captured the stability of trust-based volunteer–older adult relationships.

Informational continuity (digital literacy) was measured by smartphone ownership and the ability to independently use digital health-related functions, including appointment booking, QR code scanning, and teleconsultation, indicating potential gaps in information access and handover safety.

Managerial continuity (institutional resource availability) was assessed by reported access to community-based institutional resources formally provided by the health or social care system (e.g., community health clinics and counseling rooms). In accordance with regulatory boundaries, volunteers did not provide medical or counseling services; thus, this indicator reflected system-level coordination rather than volunteer-delivered clinical care.

Additional measures included service willingness, utilization within the past 12 months, satisfaction, and unmet needs.

#### Sociodemographic and health covariates

In addition to continuity-related indicators, sociodemographic and health-related variables were collected to characterize the study population and account for structural factors that may shape service access and experience. These included age, gender, educational attainment, monthly income, living arrangement, self-rated health, and the presence of chronic conditions (e.g., hypertension, diabetes, and cardiovascular disease).

These variables were treated as contextual or control factors rather than indicators of continuity itself. In quantitative analyses, they were used to adjust regression models and to examine how underlying health status and social position condition the manifestation of continuity mechanisms.

#### Data collection

Data were collected using a hybrid approach. Trained public health graduate students administered structured face-to-face interviews, while participants with sufficient digital literacy completed secure online questionnaires via Wenjuanxing^®^ (www.wjx.cn*).* For participants with cognitive or functional limitations, proxy respondents—defined as caregivers providing ≥ 4 h of daily care—were interviewed using a standardized protocol. Where available, responses were cross-validated against service or health records [26].

#### Statistical analysis

Quantitative analyses were conducted using SPSS version 21.0 (IBM Corp.). Descriptive statistics summarized sample characteristics. Binary logistic regression models examined two outcomes: (1) service utilization, analyzed across the full sample (*n* = 880) to identify barriers to access; and (2) service satisfaction, analyzed only among respondents who had used volunteer-supported eldercare services in the previous 12 months (*n* = 409), ensuring that satisfaction outcomes reflected direct service experience rather than hypothetical perceptions.

### Qualitative phase

#### Sampling and participants

Between May and July 2024, purposive sampling was used to recruit three participant groups (*N* = 21): eight older adults actively using volunteer-supported eldercare services; seven Red Cross eldercare staff with at least two years of relevant experience; and six community volunteers with a minimum of six months of continuous service. Episodic volunteers were excluded. Older adult participants were drawn from the quantitative survey cohort, while volunteers and staff were recruited from corresponding community settings to support multi-level analysis. Data saturation—defined as fewer than 5% new codes across three consecutive interviews [27]—was reached after 21 interviews.

#### Data collection

Semi-structured interview guides were informed by preliminary quantitative findings and pilot-tested prior to use. The interview guide covered themes related to service experiences, continuity challenges, and suggestions for improvement (see Supplementary Table S1 for the complete interview protocol). Interviews lasting 30–60 minutes were conducted in Mandarin in participants’ homes or community centers. Written or thumbprint-informed consent was obtained from all participants; for individuals with limited literacy, an independent witness was present [28].

#### Qualitative analysis

A hybrid inductive–deductive thematic analysis was conducted [29]. Initial coding was guided by Haggerty et al.’s four continuity dimensions, while allowing for the emergence of additional themes. Two researchers independently coded transcripts using NVivo version 12.0, with discrepancies resolved through discussion and consensus. Matrix coding queries facilitated systematic comparison across participant groups and thematic categories [29, 30]. Themes that did not align with the initial framework were retained and analytically examined to avoid forced categorization.

### Mixed-Methods integration

Mixed-methods integration followed the Pillar Integration Process (PIP) framework [16]. Quantitative and qualitative findings were systematically aligned to identify convergence, divergence, and complementarity across datasets. Joint displays were used as analytic devices rather than descriptive summaries, explicitly linking quantitative indicators (e.g., volunteer turnover) with corresponding qualitative narratives (e.g., erosion of trust). Relational continuity mechanisms were examined by triangulating turnover indicators with accounts of relationship instability. Informational continuity was explored by contextualizing measures of digital literacy within narratives describing challenges in information access and handover. Interactions between functional and managerial continuity were examined by mapping the quantitative “utilization gap” onto qualitative accounts of the “grey zone” of unmet needs shaped by institutional arrangements.

### Ethical considerations

Ethical approval was obtained from the Institutional Review Board of Shanghai General Hospital (Protocol No. 2025 − 193). All procedures complied with the Declaration of Helsinki (2013 revision) [31]. Written or thumbprint-informed consent was obtained from all participants, with an independent witness present for individuals with limited literacy. No financial incentives were provided. Audio recordings were deleted within 48 h of transcription, and de-identified data were stored on password-protected institutional servers.

## Results

### Quantitative findings

#### Demographic and health characteristics

Of the 880 participants, 58.2% were female. Nearly half (49.3%) were aged 60–69 years, 39.0% were 70–79 years, and 11.7% were aged ≥ 80 years. The mean age was 68.9 years (SD = 4.97; range = 64.5–82.5) (Table 1; Supplementary Table S2). Most were functionally independent in activities of daily living (ADL) (87.6%), although 71.5% reported at least one chronic condition, most commonly hypertension (53.4%) and diabetes (33.3%). Digital illiteracy affected 62.0% overall, rising sharply with age (43.1% among those 60–69 years, 77.8% among those 70–79 years, and 89.3% among those ≥ 80 years). Among service users, more than one-third (35.7%) experienced high volunteer turnover—defined as three or more replacements within six months.

**Table 1:** Demographic and health characteristics of participants (*n* = 880)

Characteristic	Category	*n* (%)
Gender	Female	512 (58.2)
	Male	368 (41.8)
Age group (years)	60–69	434 (49.3)
	70–79	343 (39.0)
	≥ 80	103 (11.7)
ADL^a^ status	Independent	771 (87.6)
	Partially/Fully dependent	109 (12.4)
Digital literacy	Digitally literate (able)	334 (38.0)
	Not digitally literate (unable)	546 (62.0)
Digital literacy by age	60–69 years (not literate)	187 (43.1)
	70–79 years (not literate)	267 (77.8)
	≥ 80 years (not literate)	92 (89.3)
Volunteer turnover ^b^	≥ 3 changes in past 6 months	146 (35.7)
	≤ 2 changes in past 6 months	263 (64.3)
Chronic conditions	≥ 1 chronic condition	629 (71.5)
	Hypertension	470 (53.4)
	Diabetes	293 (33.3)
	Cardiovascular disease	98 (11.1)
	Cerebrovascular disease	102 (11.6)
	Cancer or other	21 ( 2.4)

Note: ^a^ADL = activities of daily living, ^b^ Volunteer turnover was analyzed only among service users (*n* = 409); percentages for these sub-categories are calculated based on this *n* = 409 denominator

#### Service utilization and unmet needs

Although willingness to use volunteer-supported eldercare services was nearly universal (96.3%), actual utilization in the past year was limited to 46.5% (Table 2; Supplementary Table S3). Among service users (*n* = 409), just over half (51.3%) were satisfied, 16.4% were dissatisfied, and 32.3% were neutral or non-responsive. The most frequently reported unmet needs were rehabilitation training (45.9%) and basic medical consultation (40.8%). Fewer than 40% reported access to community-based institutional resources, including a nearby health clinic (37.7%) or counseling room (35.9%). Participants could report more than one unmet service need.

**Table 2:** Service utilization and unmet service needs (*n* = 880)

Variable	Category	*n* (%)
Willingness to use services	Willing	847 (96.3)
Service utilization	Used services in past 12 months	409 (46.5)
User satisfaction ^a^	Satisfied	210 (51.3)
	Dissatisfied	67 (16.4)
	Neutral / No response	132 (32.3)
Unmet service needs ^b^	Rehabilitation training	404 (45.9)
	Basic medical consultation	359 (40.8)
Community resource access	Health clinic	332 (37.7)
	Counseling room	316 (35.9)

Note: ^a^ Percentage based on service users (*n* = 409), ^b^ Multiple responses allowed for unmet service needs

#### Predictors of service outcomes

The four continuity dimensions were applied as an a priori analytic framework but were not assumed to operate as parallel independent predictors within a single quantitative model. Consistent with the study’s multilevel conceptualization, continuity was treated as a system-level property, while individual-level outcomes such as service dissatisfaction were analyzed as downstream manifestations of specific continuity mechanisms rather than direct measures of continuity.

Among service users (*n* = 409), binary logistic regression identified volunteer turnover as the strongest statistical correlate of dissatisfaction. Participants experiencing three or more volunteer replacements within six months were significantly more likely to report dissatisfaction (aOR = 2.31, 95% CI: 1.83–3.14; *p* < 0.001), indicating that relational instability is associated with poorer service experience.

At the access stage, informational barriers were prominent. Across the full sample (*n* = 880), digital illiteracy was significantly associated with lower service utilization (χ² = 18.261; *p* < 0.001). In contrast, health-related factors were more strongly associated with dissatisfaction among service users, including ADL dependence (*p* = 0.009), poor self-rated health (*p* = 0.005), and hypertension (*p* = 0.003). Diabetes was associated with reduced service utilization (aOR = 0.54, 95% CI: 0.31–0.93; *p* = 0.001) (Table 3).

Taken together, these findings suggest that relational continuity operates as a mechanism-level correlate of dissatisfaction among users, whereas informational continuity functions primarily as a structural barrier to service utilization.

**Table 3:** Factors associated with service utilization (*n* = 880) and service dissatisfaction among users (*n* = 409)

Factor	Comparison Group	Test Type	Value (χ²) / aOR (95% CI)	*p*-value
Volunteer Turnover (Relational Continuity)	≥ 3 vs. ≤2 replacements (Past 6 months)	Logistic Regression^a^	aOR = 2.31 (1.83–3.14)	< 0.001
Diabetes	Yes vs. No	Logistic Regression^a^	aOR = 0.54 (0.31–0.93)	0.001
Digital Literacy (Informational Continuity)	Unable vs. Able	Chi-square	χ² = 18.261	< 0.001
Cancer/Other	Yes vs. No	Chi-square	χ² = 17.533	0.002
Hypertension	Yes vs. No	Chi-square	χ² = 16.286	0.003
Cerebrovascular Disease	Yes vs. No	Chi-square	χ² = 9.792	0.044
Self-Rated Health	Poor vs. Good	Chi-square	χ² = 14.994	0.005
ADL Status	Independent vs. Partially/Fully dependent	Chi-square	χ² = 13.582	0.009
Education Level	College or above vs. Below	Chi-square	χ² = 5.509	0.138
Age Group	≥ 80 vs. 60–79	Chi-square	χ² = 13.089	0.109

Note: Factors for service utilization (*n* = 880) were tested via Chi-square; predictors for service dissatisfaction (*n* = 409) were analyzed via binary logistic regression

^a^ Adjusted for age, gender, and monthly income

*CI *confidence interval

### Qualitative findings

Consistent with the study aims, the qualitative phase focused on examining how barriers identified in the survey were experienced and interpreted across multiple system levels.

#### Participant characteristics

The qualitative sample comprised 21 participants, including eight older adults aged 74–82 years, seven staff members involved in the coordination of volunteer-supported eldercare services (primarily affiliated with the Red Cross), and six community volunteers (excluding episodic participation). Most elder adult participants had not completed high school, whereas 85.7% of staff and volunteers held at least a bachelor’s degree (Supplementary Table S4).

Consistent with the quantitative phase, ethical safeguards were strictly applied. Proxy interviews were conducted when participants had cognitive or functional limitations, and all interviews followed witnessed consent procedures. Older adult participants were recruited from the survey cohort, while staff and volunteers were recruited from corresponding community settings (see Methods), ensuring methodological coherence across study phases.

#### Multilevel barriers undermining continuity

Thematic analysis identified co-occurring barriers across policy, organizational, service, and individual levels. Rather than isolated problems, participants described these barriers as interconnected conditions shaping how volunteer-supported eldercare services were delivered, experienced, and sustained.

##### Policy level

perceived institutional constraints (functional and managerial discontinuity). Participants frequently described LTCI eligibility thresholds as creating a “grey zone” for moderately disabled older adults, limiting access to formal services and shifting expectations toward volunteer-supported support.

*“The LTCI policy only helps the totally disabled. But what about folks like me who still need a hand?” (N16)*.

These accounts reflect perceived misalignment between policy eligibility and functional needs, alongside ambiguity about responsibility allocation across sectors.

##### Organizational level

managerial fragmentation and relational instability. Participants highlighted the absence of standard operating procedures (SOPs) and siloed governance between medical institutions and volunteer organizations, which weakened task allocation, supervision, and coordination. Organizational ambiguity was also linked to unstable engagement and relational disruption.

*“Some volunteers just show up. Residents don’t even know who they are.” (N2)*.

##### Service level

functional mismatch and informational gaps. Volunteer-supported services were often described as task-focused and limited in addressing psychosocial or early health concerns.

*“Haircuts and meals are nice*,* but I need someone to talk to.” (N14)*.

Weak feedback pathways were described as further constraining informational continuity, as volunteers lacked formal channels to communicate observations to healthcare professionals.

##### Individual level

informational and relational erosion in everyday encounters. Participants described digital illiteracy among the oldest-old and frequent volunteer replacements as eroding informational access, perceived safety, and relational trust. Even when volunteers used informal compensatory practices, these were viewed as fragile substitutes for reliable information systems.

*“The older woman had a medication allergy that wasn’t documented—luckily*,* the volunteer asked again.” (N16)*.

these were viewed as fragile substitutes for reliable information systems.

**Fig. 1:**
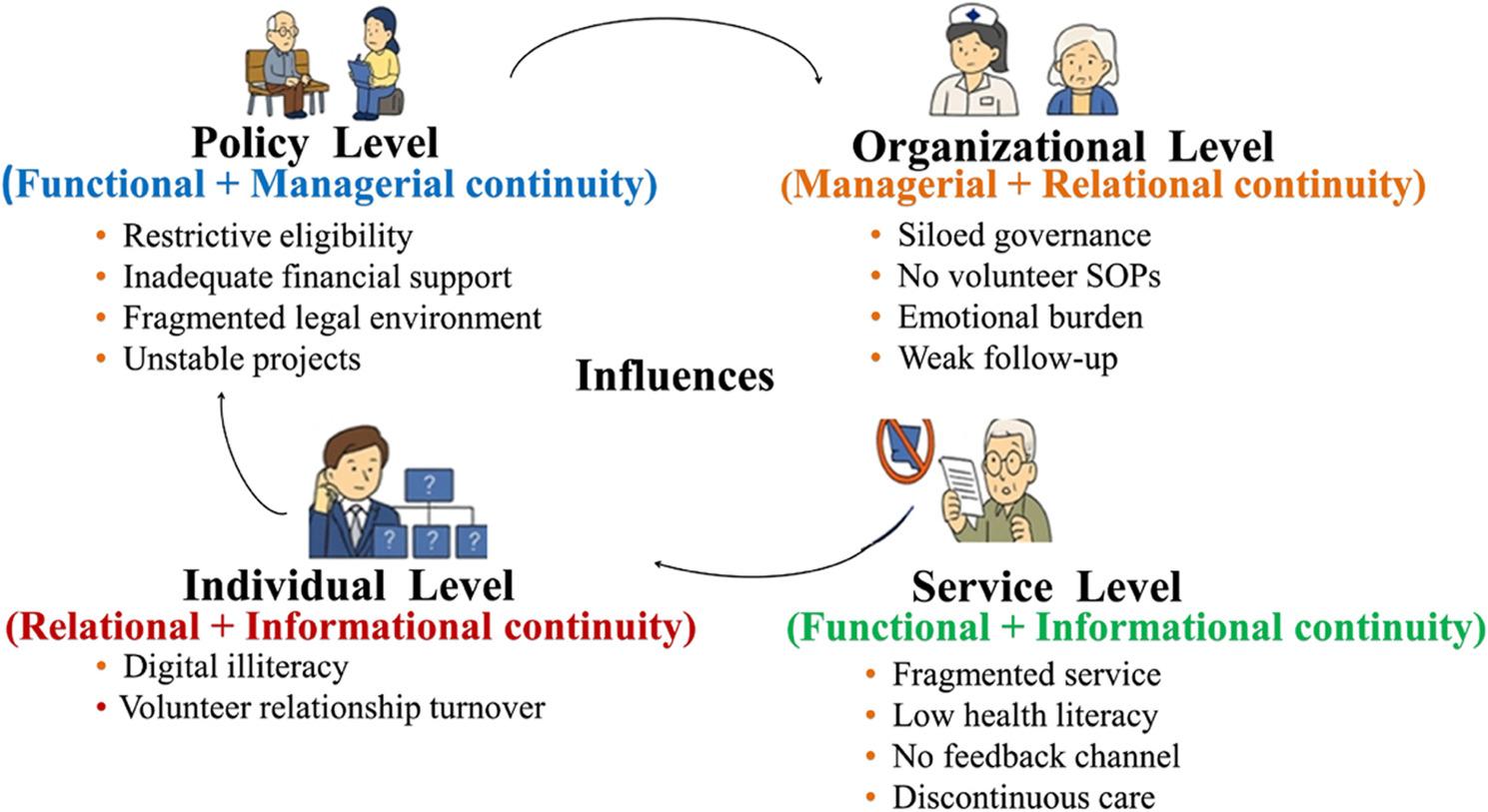
Interlocking Mechanisms Undermining Care Continuity Across Policy, Organizational, Service, and Individual Levels. Four interconnected levels—policy (functional + managerial), organizational (managerial + relational), service (functional + informational), and individual (relational + informational)—generate mutually reinforcing barriers undermining continuity. Elements are derived from thematic analysis (see Table 4 for detailed categories, codes, and illustrative quotations); continuity domains indicate analytic interpretation rather than discrete causal attribution

Illustrative quotations and their analytic mapping to the continuity dimensions are presented in Table 4, and Fig. 1 summarizes the interlocking mechanisms across system levels.

These accounts reflect how institutional eligibility thresholds were perceived to constrain functional continuity, while also generating managerial ambiguities regarding responsibility allocation between formal systems and volunteer-supported arrangements.

**Table 4:** Barriers to integration of Volunteer-Supported eldercare services into Community-Based Systems, mapped by continuity dimension

Level	Category	Codes	Illustrative quotes	Theoretical–continuity linkage
Policy	Restrictive eligibility in national policies	LTCI excludes older adults with moderate disabilities, shifting unmet needs to volunteer-supported eldercare services	“The LTCI policy only helps the totally disabled. But what about folks like me who still need a hand?” (N16); “My memory’s getting worse, but since I’m not ‘Level 3,’ I get nothing.” (N15)	Institutional exclusion and equity gaps (Functional continuity)
	Inadequate and inconsistent financial support	Funding for volunteer-supported eldercare services is symbolic or fragmented	“The Red Cross gives Renminbi (RMB) 700 a year—how far does that go? Not even enough for diapers.” (N3); “Unless you’re 90 or penniless, forget about help from the government.” (N20)	Resource fragmentation and financial unsustainability (Functional continuity)
	Fragmented legal and policy environment	Lack of unified guidelines or clear responsibilities for integrating volunteer-supported eldercare services	“Sometimes it’s health, sometimes civil affairs—no one really takes charge.” (N3); “If something happens, who’s responsible? Volunteers have no protection.” (N1)	Governance ambiguity and legal accountability void (Managerial continuity)
	Unstable project-based operations	Short-term grants undermine sustainability; participation of volunteer-supported eldercare services tied to project cycles	“Volunteers come for a project, then disappear. No one sticks around.” (N7)	Volunteer turnover and temporal instability (Managerial continuity)
Organizational	Siloed governance and poor coordination	Lack of collaboration between Red Cross, clinics, and social workers in delivering volunteer-supported eldercare services	“Each group does their own thing. The health center doesn’t talk to the Red Cross.” (N4); “Nobody organizes them together—it’s all scattered.” (N17)	Organizational fragmentation and lack of cross-sector integration (Managerial continuity)
	Absence of SOPs for volunteer integration	Lack of standard procedures to integrate volunteer-supported eldercare services into multidisciplinary teams	“Some volunteers just show up. Residents don’t even know who they are.” (N2); “They want to help more, but no one tells them how.” (N20); “I was asked to help an older man with diabetes, but no one told me what to do or what to watch out for.” (N9)	Absence of SOPs and role ambiguity; training deficits (Managerial continuity)
	Emotional burden and lack of support	Volunteers delivering eldercare services face emotional distress without structured debriefing	“I visited him at home and noticed something was wrong—he looked dazed, barely responded. But I had no one to report it to. Later, I found out he passed away. I couldn’t sleep for days.” (N10)	Compassion fatigue and emotional oversight (Relational continuity)
	Lack of continuity and follow-up	Screenings or visits by volunteer-supported eldercare services lack sustained follow-up or feedback	“They told me to go see a doctor but didn’t explain anything.” (N18); “I got a report, but who knows what the numbers mean?” (N21); “One visit, then nothing. No plan, no calls.” (N15)	Disrupted relational continuity and weak follow-up (Relational continuity)
Service	Fragmented and basic offerings	Volunteer-supported eldercare services focus on instrumental tasks and neglect psychosocial needs	“Haircuts and meals are nice, but I need someone to talk to.” (N14)	Superficial coverage and unmet psychosocial needs (Functional continuity)
	Limited health literacy	Older adults receiving volunteer-supported eldercare services do not understand medical reports	“I get those checkup papers every year—I don’t even open them.” (N20)	Health literacy deficits and informational discontinuity (Informational continuity)
	Disconnected communication pathways	No feedback loop between volunteers delivering eldercare services and health professionals	“I noticed she was depressed and skipping meds, but I didn’t know who to report that to.” (N12)	Information discontinuity and feedback loop deficits (Informational continuity)
	Service discontinuity resulting from project-based operations	Follow-up care from volunteer-supported eldercare services ceases after project funding ends	“They checked my blood pressure once—after that, no follow-up at all.” (N18)	Episodic service provision and weak follow-up mechanisms (Functional continuity)
Individual	Digital illiteracy	Oldest-old recipients of volunteer-supported eldercare services lack access to digital tools	“I can’t see my health records—so I don’t know if my allergies or medications are tracked.” (N15)	Digital literacy deficits and safety risks (Informational continuity)
	Inconsistent volunteer relationships	High turnover in volunteers-supported eldercare services undermine trust	“Transient volunteers? No point confiding.” (N18); “The older woman had a medication allergy that wasn’t documented—luckily, the volunteer asked again.” (N16)	Relational attrition and trust deficits (Relational continuity)

Note: Terminology has been standardized to “volunteer-supported eldercare services” for consistency across the manuscript. “Digital illiteracy” is used to denote the inability of older adults to independently use digital health tools (e.g., appointment booking, QR code scanning, teleconsultation), as defined in the Methods section. The continuity domains indicate analytic mapping of qualitative themes to the theoretical framework, rather than discrete causal attribution, and are used to denote the primary dimension through which each barrier was interpreted. For a complete set of illustrative quotations systematically mapped to all continuity dimensions, see Supplementary Table S5

*LTCI* Long-term care insurance, *SOPs* Standard operating procedures, *RMB* Renminbi

Four interconnected levels—policy (functional + managerial), organizational (managerial + relational), service (functional + informational), and individual (relational + informational)—generate mutually reinforcing barriers undermining continuity. Elements are derived from thematic analysis (see Table 4 for detailed categories, codes, and illustrative quotations); continuity domains indicate analytic interpretation rather than discrete causal attribution.

### Mixed-methods integration

**Fig. 2:**
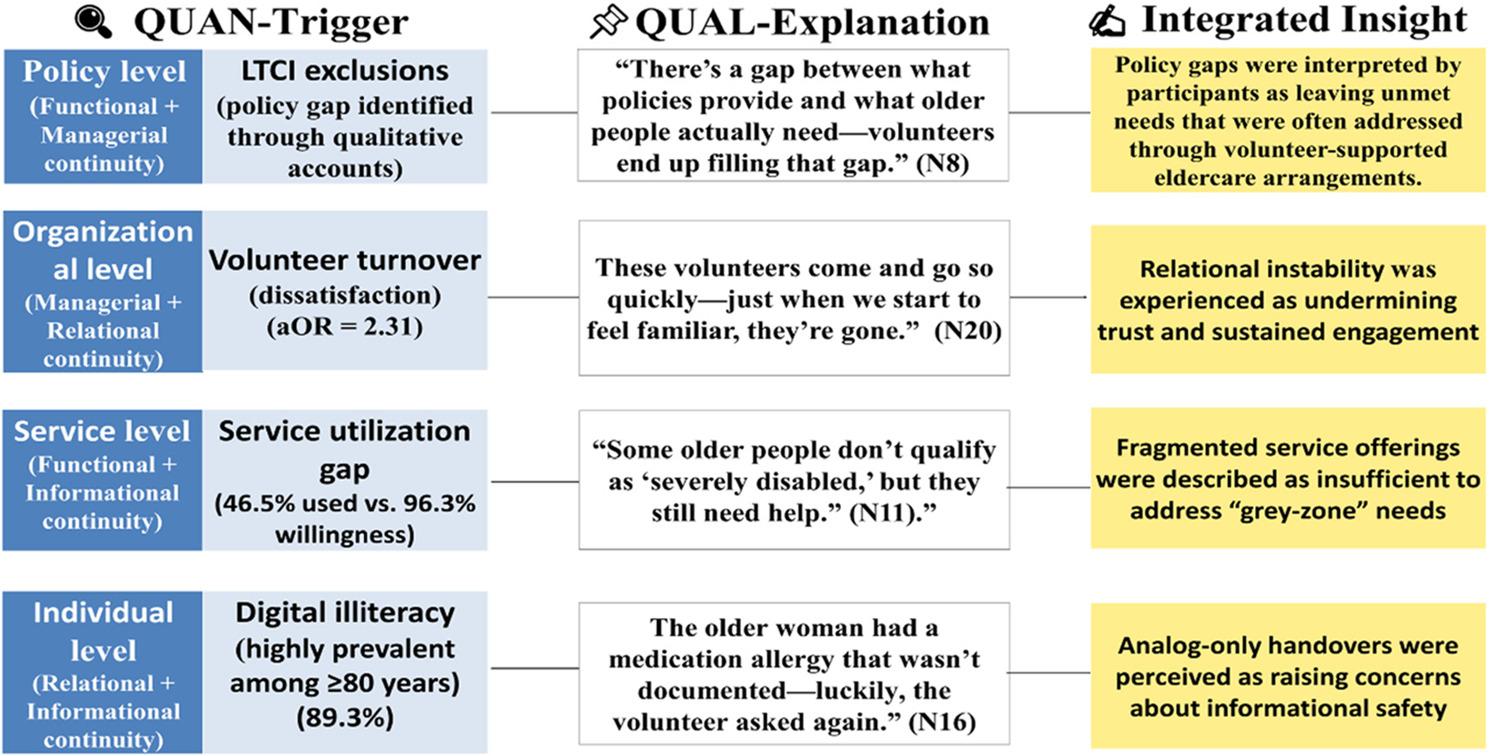
Joint Display of QUAN→QUAL Integration Pathway on Volunteer–Supported Eldercare Services Gaps. This framework illustrates how quantitative disparities (e.g., service underutilization, volunteer turnover, LTCI exclusions, and digital illiteracy) correspond to continuity disruptions across policy, organizational, service, and individual levels. Qualitative explanations contextualize these patterns by elucidating perceived mechanisms and lived experiences, rather than serving as causal validation. The integrated insights reflect analytic interpretation of participants’ accounts rather than causal attribution

Consistent with an explanatory sequential design, integration was intended to generate mechanism-oriented interpretation rather than to establish causal correspondence between datasets. Integration of quantitative and qualitative findings identified four interrelated discontinuities through which barriers to volunteer-supported eldercare services were experienced and sustained: (1) policy exclusion (LTCI-related “grey zone” needs); (2) organizational instability (high volunteer turnover and lack of SOPs); (3) service fragmentation (narrow offerings and weak coordination); and (4) individual digital barriers (limited digital literacy and unsafe handovers) (Table 5; Fig. 2).

Quantitative signals—particularly the willingness–utilization gap (96.3% vs. 46.5%) and the association between dissatisfaction and volunteer turnover (aOR = 2.31)—were explained through qualitative accounts that clarified mechanisms and contextual conditions. Joint displays were used to link statistical patterns to stakeholder narratives and to support mechanism-oriented interpretation rather than causal validation.

#### Explanatory synthesis

Guided by an explanatory QUAN→QUAL integration framework (Fig. 2), observed discontinuities were analytically aligned with the four continuity domains: (1) functional continuity—limitations in the scope of basic and psychosocial support; (2) relational continuity—erosion of trust associated with frequent volunteer turnover; (3) informational continuity—documentation gaps, digital exclusion, and weak feedback loops; and (4) managerial continuity—fragmented governance arrangements and project-based instability.

Joint displays (Table 5) were used to synthesize quantitative indicators with qualitative narratives, generating mechanism-oriented insights into how these discontinuities co-occur and are sustained across policy, organizational, service, and individual levels. This integration was intended to support analytic interpretation rather than to imply discrete causal pathways. Continuity domains therefore reflect integrative mapping across datasets, not independent statistical constructs. Quantitative triggers indicate points of analytic entry rather than independent predictors of continuity.

**Table 5:** Joint display of QUAN→QUAL integration pathway on Volunteer-Supported eldercare services gaps

Level	Quantitative Trigger (QUAN)	Qualitative Explanation (Quote)	Integrated Insight	Policy/Practice Implication
Policy	LTCI exclusions (QUAL-detected policy gap)	“There’s a gap between what policies provide and what older people actually need—volunteers end up filling that gap.” (N8)	Policy–service dissonance shifts unmet needs to volunteer-supported eldercare services, particularly excluding moderately disabled older adults.	Expand LTCI eligibility to include moderate disability; pilot reimbursement for volunteer-supported essential care
Organizational	Volunteer turnover → ↑ dissatisfaction (aOR = 2.31; 95% CI: 1.83–3.14)	“These volunteers come and go so quickly—just when we start to feel familiar, they’re gone.” (N20)	Relational instability undermines trust and engagement in volunteer-supported eldercare services	Develop retention incentives; embed volunteers within interdisciplinary teams; establish SOPs for continuity
Service	Utilization gap: 46.5% used vs. 96.3% willingness	“Some older people don’t qualify as ‘severely disabled,’ but they still need help.” (N11)	Fragmented service offerings fail to cover “grey-zone” needs, including gaps in psychosocial support.	Build community-level care coordination and navigation mechanisms; enable flexible pathways for access to non-LTCI-covered services
Individual	Digital illiteracy highly prevalent among ≥ 80 years (89.3%)	“The older woman had a medication allergy that wasn’t documented—luckily, the volunteer asked again.” (N16)	Low digital literacy and analog-only handovers compromise informational safety and continuity during care transitions.	Create community-based digital literacy hubs; adopt hybrid (paper–digital) handover tools; design elder-friendly information systems

## Discussion

### Study context

In this study, continuity is conceptualized as an emergent system property rather than a directly measurable individual outcome. In volunteer-supported eldercare service systems, continuity remains an organizational and system-level property, while being ultimately manifested through older adults’ lived experiences of access, trust, and informational safety. This explanatory sequential mixed-methods study examined systemic barriers to integrating volunteer-supported eldercare services in urban China, a setting undergoing rapid demographic transition. Quantitative surveys identified utilization patterns, while qualitative interviews elucidated underlying mechanisms. By applying the Pillar Integration Process [16] and joint displays [17, 32], we achieved coherent QUAN–QUAL integration across policy, organizational, service, and individual domains. Importantly, this approach also aligns with global scholarship that emphasizes connecting numeric trends with narrative insights to capture the complexity of volunteer contributions to health and social care systems [2, 33].

### Key quantitative findings

The observed willingness–utilization gap highlights an important structural tension in volunteer-supported eldercare services: although 96.3% of older adults expressed willingness to use volunteer-supported services, only 46.5% had done so. Among the oldest-old (≥ 80 years), digital illiteracy (89.3%) severely restricted access to booking, follow-up, and teleconsultation [34]. In addition, volunteer turnover emerged as the strongest correlate of dissatisfaction (aOR = 2.31, 95% CI: 1.83–3.14), highlighting relational discontinuity. This parallels findings from the UK’s Royal Voluntary Service, where stable volunteer–client relationships underpin trust [3]. Collectively, these quantitative patterns suggest that service utilization may depend less on client willingness than on systemic capacity to sustain functional, relational, informational, and managerial continuity [23, 24, 35, 36].

### Qualitative mechanisms and systemic interactions

The qualitative analysis contextualized these findings and illuminated mechanisms that reinforced the observed patterns. Specifically:

#### Policy–service dissonance

Long-Term Care Insurance (LTCI) eligibility excluded many moderately disabled older adults, shifting unmet “grey-zone” needs to volunteers, echoing gaps reported in Nordic contexts [5].

#### Relational instability

High turnover and lack of Standard Operating Procedures (SOPs) disrupted trust and continuity, in contrast with Denmark’s stable volunteer–elder pairing models [5].

#### Digital–informational rupture

Digital illiteracy among the oldest-old heightened dependence on volunteers for information transfer, raising safety concerns. Singapore’s Silver Infocomm Initiative shows how structured literacy programs can mitigate this barrier [34].

#### Governance fragmentation

Absence of a coordinating body hindered collaboration among volunteers, clinicians, and social workers, consistent with global research on integration challenges [2, 33].

These mechanisms were mutually reinforcing, producing a self-perpetuating cycle of discontinuity that weakened system resilience. Importantly, while some participants valued volunteers as a buffer against system gaps, others cautioned against over-reliance on unpaid labor as a substitute for structural reform, echoing international critiques of fragmented care models [2, 33].

### International comparisons and lessons

International experience underscores that volunteer integration is most effective when institutionally embedded, financially supported, and professionally regulated. The UK’s NHS care navigation teams exemplify structured coordination [3], while Denmark’s long-term volunteer–elder pairings highlight the role of relationship stability [5]. Singapore’s community-based digital literacy hubs address technological barriers [34], and the stipend-supported US Foster Grandparent and Senior Companion programs demonstrate how financial incentives sustain engagement [37]. Australia’s Community Visitors Scheme incorporates protective manuals and risk management protocols to safeguard practice [4].

Beyond program structure, these models emphasize volunteer-supported eldercare services and protection. U.S. volunteers receive stipends, insurance, and regular training [37]; Nordic countries provide continuous supervision and competency development [5]; and Australia enforces standardized safety procedures [4]. Collectively, these examples illustrate that sustainable engagement requires not only volunteer–client matching but also institutionalized training, financial incentives, and safeguards.

Compared with these structured approaches, China’s volunteer-supported eldercare services remains heavily dependent on unpaid labor with limited protections, raising concerns regarding sustainability, equity, and burnout. Taken together, international cases reveal both common principles—stable financing, legal safeguards, professional oversight—and contextual adaptations, such as digital inclusion or culturally embedded pairing systems.

However, transferring these models to China requires adaptation to local sociocultural and governance contexts, where residents’ committees and state–society relations play distinctive roles. While these discontinuities highlight structural challenges, they also reveal entry points for reform—for example, adapting digital literacy hubs or long-term pairing models could directly target the discontinuities identified.

### Policy and practice implications

Bridging the willingness–utilization gap requires reforms across multiple levels:

#### Policy

Expand LTCI eligibility to include moderate disability and pilot reimbursement for essential non-clinical volunteer support [2, 5].

#### Organizational

Integrate volunteers into interdisciplinary teams, establish SOPs, and provide retention incentives to stabilize volunteer–client pairings [3, 4, 37].

#### Service

Develop care navigation roles to address “grey-zone” needs between medical coverage and daily living support [36].

#### Individual

Launch targeted digital literacy programs for the oldest-old, supported by hybrid paper–digital systems to safeguard informational continuity [34].

Importantly, volunteers should complement—not replace—professional services. Over-reliance on unpaid labor risks institutional inertia and widening inequities if structural reforms are not implemented in parallel [2, 33, 37]. In the short term, retention incentives and digital literacy programs may be the most feasible, whereas in the long term, expanding LTCI eligibility and institutionalizing volunteer integration will be critical.

### Methodological and theoretical contributions

This study illustrates the utility of explanatory sequential designs in health systems research. Quantitative anomalies (e.g., high willingness but low utilization) guided qualitative exploration, while qualitative narratives clarified systemic mechanisms.

The findings also extend Haggerty’s continuity framework beyond clinical care to volunteer-integrated social services. Functional, relational, informational, and managerial continuity proved equally relevant in non-clinical contexts [23, 24]. Discontinuities—including policy–service misalignment, volunteer turnover, digital illiteracy, and governance fragmentation—were shown to undermine resilience. Thus, this study contributes empirically by documenting systemic discontinuities in urban China and conceptually by validating the cross-sectoral utility of the continuity framework, bridging perspectives from health policy, sociology, and gerontology.

### Strengths and limitations

Although this study was conducted in two highly aged districts of Shanghai, the mechanisms identified—particularly digital exclusion, volunteer turnover, and governance fragmentation—are likely to be relevant to other rapidly aging urban settings facing similar pressures, albeit with context-specific manifestations. Strengths of this study include the integration of quantitative and qualitative data and a focus on an urban context experiencing advanced aging dynamics.

Several limitations should be acknowledged. First, potential recall bias may have affected self-reported survey responses. Second, the findings may have limited generalizability to rural or less-developed settings. Third, while volunteer turnover emerged as a key mechanism of relational discontinuity, this study did not quantitatively assess volunteer-level psychological stress, workload, or emotional burden. These unmeasured factors are likely to shape the stability and quality of volunteer–older adult relationships and may contribute to relational continuity disruptions beyond organizational arrangements alone.

Finally, as a cross-sectional study, causal inferences are restricted. Longitudinal or intervention-based designs that explicitly incorporate volunteer workload, stress, and support mechanisms are needed to assess how relational continuity can be sustained over time.

## Conclusions

Sustainable volunteer-supported eldercare services in China requires systemic continuity across policy, organizational, service, and individual levels. Institutional integration, cross-sector collaboration, and robust information systems are essential to move from fragmented, ad hoc models toward person-centered, resilient care [2–5, 23, 24, 33, 36, 37]. Future research should explore scalability and long-term effects across diverse urban and rural settings. Beyond China, these findings offer transferable lessons for other rapidly aging societies confronting similar challenges.

## Supplementary Information

Supplementary Material 1.

## Data Availability

The data that support the findings of this study are available from the corresponding author upon reasonable request.

## Abbreviations

ADL: Activities of Daily Living
aOR: Adjusted Odds Ratio
CI: Confidence Interval
CVI: Content Validity Index
LTC: Long-Term Care
LTCI: Long-Term Care Insurance
GRAMMS: Good Reporting of a Mixed Methods Study
OR: Odds Ratio
PIP: Pillar Integration Process
QUAN: Quantitative Component
QUAL: Qualitative Component
RMB: Renminbi
Red Cross: Red Cross Society of China
SD: Standard Deviation
SOP: Standard Operating Procedure
SRQR: Standards for Reporting Qualitative Research
