# Supplementary material for: Challenges to continuity of care in volunteer-integrated services for older adults: a mixed-methods study in urban China
Source: BMC Geriatr. 2026 Feb 11;26:362. doi: 10.1186/s12877-026-07115-4 (PMC12998205; doi:10.1186/s12877-026-07115-4)
Supplement: Supplementary file 1 — Supplementary Material 1. [file 12877_2026_7115_MOESM1_ESM.doc]

**Supplementary Table**
These supplementary materials provide extended methodological detail and expanded results, complementing but not duplicating the main text Table S1–5.

- **Supplementary Table S1.** Semi-structured interview guide used in the qualitative phase (Methods).
- **Supplementary Table S2.** Extended demographic and health characteristics of survey participants (complements Table 1).
- **Supplementary Table S3.** Detailed service utilization, satisfaction, and community resource access (complements Table 2).
- **Supplementary Table S4.** Characteristics of the qualitative sample (n = 21), supplementing participant descriptions in the Results.
- **Supplementary Table S5.** Expanded illustrative quotations mapped to Haggerty et al.’s continuity framework, supporting analyses summarized in Tables 4 and 5.

**Supplementary Table S1: Qualitative Interview Guides for Participants in Volunteer-Supported Eldercare Services**

| **Continuity Dimension / Core Focus** | **Older Adults** | | **Red Cross Eldercare Staff** | **Community Volunteers** |
| --- | --- | --- | --- | --- |
| Functional Continuity Service linkage across time and settings | | 1. Have volunteer changes ever disrupted your medical care? Please describe. | 1. How do you maintain continuity of chronic disease management during volunteer turnover? | 1. What key health information must be transferred during volunteer handovers? |
|  | | 2. When **eligibility criteria** excluded certain needs (e.g., medications), how did volunteers assist? | 2. What support mechanisms are in place for older adults excluded from policy coverage? | 2. How do you support care for those not covered by current policies? |
| Relational Continuity Sustained, trust-based relationships | | 3. How long did it take to trust a new volunteer? Please share an example. | 3. What strategies foster long-term trust between volunteers and older adults? | 3. What familiar names or nicknames do care recipients use for you? How does this impact the care relationship? |
|  | | 4. Did trusting a volunteer ever help you change a health habit (e.g., taking medications regularly)? | 4. How does volunteer retention influence service quality and elder engagement? | 4. Can you describe a time when trust improved service acceptance? |
| Informational Continuity Effective communication and knowledge transfer | | 5. What health-related information do you have to repeat to each new volunteer? | 5. How are health records updated and preserved during volunteer transitions? | 5. What are your main sources of health information? Where do you encounter gaps? |
|  | | 6. Would you share your health records with a trusted volunteer? Why or why not? | 6. Are there mechanisms for cross-institutional information sharing, especially for uncovered individuals? | 6. How do you manage and protect sensitive personal health data? |
| Management Continuity Coordination and system-level support | | 7. Does your community center maintain consistent services over time? What could be improved? | 7. Which current policies are most effective in mitigating service disruption due to turnover? | 7. Does your organization provide sufficient handover support? What areas need improvement? |
|  | | 8. What additional help do you hope volunteers could provide? | 8. Do you believe a formal volunteer–elder pairing mechanism is feasible and beneficial? | 8. What additional training or authority would help you assist those excluded from policy support? |

*Note: “Volunteers” are defined as* *semi-formal actors delivering volunteer-supported eldercare services within the community-based eldercare system.*

**Supplementary Table S2: Quantitative Participant demographic and Health Characteristics (n=880)**

| **Variable** | **Category** | **n (%)** |
| --- | --- | --- |
| Gender | Female | 512 (58.2) |
|  | Male | 368 (41.8) |
| Age group(years) | 60–69 | 434 (49.3) |
|  | 70–79 | 343 (39.0) |
|  | ≥80 | 103 (11.7) |
| Education level | Junior high or below | 272 (30.9) |
|  | High school/Technical | 403 (45.8) |
|  | College or above | 205 (23.3) |
| Marital status | Married | 740 (84.1) |
|  | Divorced | 112 (12.7) |
|  | Widowed/Single | 28 (3.2) |
| Monthly income | ≤3,000 RMB | 312 (35.5) |
|  | 3,001–6,000 RMB | 409 (46.5) |
|  | >6,000 RMB | 159 (18.0) |
| Number of children | 1 | 509 (57.8) |
|  | 2 | 321 (36.5) |
|  | ≥3 | 50 (5.7) |
| Self-rated health | Poor | 629 (71.5) |
|  | Good | 251 (28.5) |
| **ADL^a^ status** | Independent | 771 (87.6) |
|  | Partially/fully dependent | 109 (12.4) |
| Digital literacy | Digitally literate (Able) | 334 (38.0) |
|  | Not digitally literate (Unable) | 546 (62.0) |
| Digital literacy by age | 60–69 years (Not literate) | 187 (43.1) |
|  | 70–79 years (Not literate) | 267 (77.8) |
|  | ≥80 years (Not literate) | 92 (89.3) |
| Volunteer turnover ^b^ | ≥3 changes in past 6 months | 146(35.7) |
|  | ≤2 changes in past 6 months | 263 (64.3) |
| Chronic conditions | ≥1 chronic condition | 629 (71.5) |
|  | Hypertension | 470 (53.4) |
|  | Diabetes | 293 (33.3) |
|  | Cardiovascular disease | 98 (11.1) |
|  | Cerebrovascular disease | 102 (11.6) |
|  | Cancer and others | 21 (2.4) |

*Note: ^a^ADL = activities of daily living. ^b^ Volunteer turnover was analyzed only among service users (n=409); percentages for these sub-categories are calculated based on this n=409 denominator. RMB* Renminbi

**Supplementary Table S3: Service Utilization and Unmet Service Needs (n = 880)**

| **Variable** | **Category** | **n (%)** |
| --- | --- | --- |
| Willingness to use services | Willing | 847 (96.3) |
|  | Not willing | 33 ( 3.7) |
| Service Utilization | Used services in past 12 months | 409 (46.5) |
|  | Did not use services in past 12 months | 471 (53.5) |
| User Satisfaction ^a^ | Satisfied | 210 (51.3) |
|  | Neutral/No response | 132 (32.3) |
|  | Dissatisfied | 67 (16.4) |
| Unmet Service Needs ^b^ | Rehabilitation training | 404 (45.9) |
|  | Basic medical consultation | 359 (40.8) |
| Community resource access | Community comprehensive center | 641 (72.8) |
|  | Elder service center | 519 (59.0) |
|  | Day care institution | 504 (57.3) |
|  | Senior care home | 451 (51.3) |
|  | Health clinic | 332 (37.7) |
|  | Meal service site | 477 (54.2) |
|  | Senior activity room | 519 (59.0) |
|  | Counseling room | 316 (35.9) |

*Note: ^a^ Percentage based on service users (n = 409). ^b^ Multiple responses allowed for unmet service needs*.

**Supplementary Table S4: Qualitative participant characteristics**

| ID | Stakeholder Type | Gender | Age | Education | ADL Status | Proxy Interview |
| --- | --- | --- | --- | --- | --- | --- |
| N1 | Staff | F | 42 | Bachelor | – | No |
| N2 | Staff | M | 49 | Bachelor | – | No |
| N3 | Staff | F | 51 | Master | – | No |
| N4 | Staff | M | 47 | Bachelor | – | No |
| N5 | Staff | F | 35 | Bachelor | – | No |
| N6 | Staff | M | 45 | Bachelor | – | No |
| N7 | Staff | F | 41 | Bachelor | – | No |
| N8 | Volunteer | F | 26 | Bachelor | – | No |
| N9 | Volunteer | M | 30 | Bachelor | – | No |
| N10 | Volunteer | F | 34 | Master | – | No |
| N11 | Volunteer | F | 28 | Bachelor | – | No |
| N12 | Volunteer | M | 31 | Bachelor | – | No |
| N13 | Volunteer | F | 29 | Bachelor | – | No |
| N14 | Older Adult | M | 74 | Junior High | Independent | No |
| N15 | Older Adult | F | 78 | Primary | Independent | No |
| N16 | Older Adult | F | 81 | Primary | Partially Dependent | Yes |
| N17 | Older Adult | M | 75 | Junior High | Independent | No |
| N18 | Older Adult | F | 76 | Junior High | Independent | No |
| N19 | Older Adult | F | 80 | Primary | Dependent | Yes |
| N20 | Older Adult | M | 77 | Primary | Independent | No |
| N21 | Older Adult | F | 82 | Junior High | Independent | No |

***Note:*** *ADL status assessed using the Katz Index of Independence in Activities of Daily Living. “Yes” under Proxy Interview indicates responses provided by a caregiver or proxy due to limited verbal capacity. Staff = Red Cross or community eldercare personnel; Volunteers = providers of volunteer-supported eldercare services.*

**Supplementary Table S5. Continuity Barriers with Illustrative Quotes and Theoretical Linkages**

| ***Continuity Dimension*** | ***Barrier*** | ***Illustrative Quote*** | ***Theoretical Linkage*** |
| --- | --- | --- | --- |
| Functional continuity | LTCI excludes moderate disabilities | “The LTCI policy only helps the totally disabled. But what about folks like me who still need a hand?” (N16) | Institutional Exclusion; Equity Gaps ^[11]^ |
|  | Insufficient and inconsistent financial support | “The Red Cross gives RMB 700 a year—how far does that go? Not even enough for diapers.” (N3) | Resource Fragmentation; Financial Unsustainability ^[11]^ |
|  | Superficial services fail to meet psychosocial needs | “Haircuts and meals are nice, but I need someone to talk to.” (N14) | Superficial Coverage; Unmet Psychosocial Needs ^[14]^ |
| Managerial continuity | Lack of unified guidelines and unclear responsibilities | “Sometimes it's health, sometimes civil affairs—no one really takes charge.” (N3) | Governance Ambiguity; Legal Accountability Void ^[23]^ |
|  | Short-term, project-based engagement of volunteers delivering volunteer-supported eldercare services | “Volunteers come for a project, then disappear.” (N7) | Volunteer Turnover; Temporal Instability ^[24]^ |
|  | Absence of SOPs for integration of volunteers into volunteer-supported eldercare services | “I was asked to help an older man with diabetes, but no one told me what to do or what to watch out for.” (N9) | Role Ambiguity; Training Deficits ^[25]^ |
| Relational continuity | Volunteer emotional burden without debriefing support | “I couldn’t sleep for days after I found out the man I visited had died. There was no one to talk to.” (N10) | Compassion Fatigue; Emotional Oversight ^[25]^ |
|  | High turnover among volunteers delivering volunteer-supported eldercare services undermines trust | “Transient volunteers? No point confiding.” (N18) | Relational Attrition; Trust Deficits ^[23]^ |
|  | Weak follow-up after initial contact | “They told me to go see a doctor but didn’t explain anything.” (N18) | Disrupted Relational Continuity; Weak Follow-Up ^[14]^ |
| Informational continuity | Health literacy deficits among older adults | “I get those checkup papers every year—I don’t even open them.” (N20) | Health Literacy Deficits; Informational Discontinuity ^[14]^ |
|  | No feedback loop between volunteers delivering volunteer-supported eldercare services and professionals | “I noticed she was depressed and skipping meds, but I didn’t know who to report that to.” (N12) | Information Discontinuity; Feedback Loop Deficits ^[25]^ |
|  | Digital illiteracy limiting access to health information | “I can’t see my health records—so I don’t know if my allergies or medications are tracked.” (N15) | Digital Literacy; Safety Risks ^[14]^ |

**Note:** N1–N7 = staff; N8–N13 = volunteers; N14–N21 = older adults. “Volunteers” are defined as semi-formal actors delivering services institutionally embedded within and coordinated with local care systems. Quotes are derived from semi-structured interviews (see Supplementary Table S4 for details). Terminology (LTCI, SOPs, digital illiteracy) and references follow the main text.
